# Morning/Evening Differences in Somatosensory Inputs for Postural Control

**DOI:** 10.1155/2014/287436

**Published:** 2014-08-18

**Authors:** Clément Bougard, Damien Davenne

**Affiliations:** ^1^Armed Forces Biomedical Research (IRBA), Vigilance Team, 91223 Brétigny-sur-Orge, France; ^2^Université Paris Descartes, Sorbonne Paris Cité, EA 7330 VIFASOM Sommeil-Fatigue-Vigilance et Santé Publique, 75181 Paris, France; ^3^Normandie University, 14032 Caen, France; ^4^Unicaen, COMETE, 14032 Caen, France; ^5^INSERM, U 1075, COMETE, 14032 Caen, France

## Abstract

The underlying processes responsible for the differences between morning and afternoon measurements of postural control have not yet been clearly identified. This study was conducted to specify the role played by vestibular, visual, and somatosensory inputs in postural balance and their link with the diurnal fluctuations of body temperature and vigilance level. Nineteen healthy male subjects (mean age: 20.5 ± 1.3 years) participated in test sessions at 6:00 a.m. and 6:00 p.m. after a normal night's sleep. Temperature was measured before the subjects completed a sign cancellation test and a postural control evaluation with eyes both open and closed. Our results confirmed that postural control improved throughout the day according to the circadian rhythm of body temperature and sleepiness/vigilance. The path length as a function of surface ratio increased between 6:00 a.m. and 6:00 p.m. This is due to a decrease in the centre-of-pressure surface area, which is associated with an increase in path length. Romberg's index did not change throughout the day; however, the spectral analysis (fast Fourier transform) of the centre-of-pressure excursions (in anteroposterior and mediolateral directions) indicated that diurnal fluctuations in postural control may occur via changes in the different processes responsible for readjustment via muscle contractions.

## 1. Introduction

Various studies reported an influence of time-of-day on postural control [1–5], even if the results were founded on various materials, experimental procedures, and evaluation criteria [6]. When external parameters were controlled, it has been shown that the greatest variations in balance capacities throughout a normal day were observed between 6:00 a.m. and 6:00 p.m. [7]. However, the underlying processes responsible for these diurnal fluctuations in postural control have not yet been clearly identified.

Balance is maintained by the continuous and effective integration of vestibular, visual, and proprioceptive information in the central nervous system (CNS) [8]. All of this sensorial information is processed in the cerebellum, enabling the centre of gravity (CG) to be supported and maintained by postural muscle contractions [9]. More precisely, the vestibulocerebellum is a cerebral structure that is involved in postural control regulation [10] and visuomotor coordination [11]. Another part of the cerebellum, called the cerebrocerebellum, is involved in the regulation of various nonmotor functions such as attention and/or cognition [12]. Moreover, various studies reported changes in cerebellum activation during sleep [13] and with increased sleepiness [14], which can impact motor activity. As a consequence, a number of studies suggested that close relationships between balance capacities and the level of sleepiness can be considered [1, 4, 6, 7].

Through the use of spectral analysis [9], the evaluation of the possible contributions of vestibular, visual, and somatosensory inputs to postural control throughout the day is of great interest. Even if there is still debate on this approach, either on the range of total frequency content [15, 16] or band-width size [15, 16], studies based on sleep deprivation paradigms (which modify the level of sleepiness/vigilance) reported that the vestibular system would be the sensory input most affected by an increase in the length of time awake [2, 4, 6]. But other studies concluded that visual input becomes less efficient with the length of time awake, or that the integration of visual information becomes deficient or slower [17].

While the postural control system appears to use distinct control strategies in the anteroposterior and mediolateral directions [18], the spectral analysis was only conducted on the global signal. Recent studies have shown that the identification of somatosensory inputs has to take into account the spectral content of postural sway on each direction separately (anteroposterior and mediolateral) [19, 20]. This is because the evolution of one sensory input can be different in each direction throughout the day. This approach can give further information on the underlying processes responsible for the previously observed changes in postural strategies by using the distribution of variability at different frequencies. While investigating time-of-day effects on postural control, one would expect that postural sway in the anteroposterior and mediolateral directions may require different processes in the morning than in the afternoon.

Furthermore, it would be interesting to test the circadian rhythms of other physiological variables such as temperature and vigilance (which are the most studied because of their repercussions on human behaviour [21]) to examine if they evolve similarly to postural control fluctuations. This would notably bring further information on possible compensatory adjustments. Temperature is often used as the gold standard test to evaluate circadian rhythmicity [21]. Various studies have shown that postural sway evolves in harmony with the temperature rhythm [22, 23], peaking in the early morning (between 5:00 a.m. and 7:00 a.m.), during the bathyphase of the body temperature rhythm [2, 22]. Temperature also influences many other physiological regulations that are involved in postural sway regulations [24]. Vigilance reflects the level of CNSs activation and the varied levels can be estimated on a continuum between wake and sleep (vice versa for sleepiness) [25]. It has been shown that postural control is modified by the level of vigilance or sleepiness [4, 6, 17, 22, 23, 26–28]. Since maintaining balance requires continuous integration of different sensory inputs, it has been proposed that vigilance impairment affects the processes of these integrations at the CNS level and, in parallel, also affects the processes underlying efficient adjustments of movements involved in postural sway regulation [17, 26, 27].

In this study, oral temperature, vigilance, and postural sway parameters have been recorded in parallel to specify (i) the link between these parameters and (ii) the role played by vestibular, visual, and somatosensory inputs in the diurnal fluctuations of postural control. For that, a frequency analysis applied on centre-of-pressure excursions has been used in the anteroposterior and mediolateral directions.

## 2. Methods and Materials

### 2.1. Subjects

Nineteen nonsmoking male subjects (age: 20.5 ± 1.3 years; body mass: 70.0 ± 6.1 kg; height: 179.0 ± 4.8 cm) participated in this study, which was granted ethical approval by the ethics committee (Comité de Protection des Personnes Nord-Ouest III, number 2007-A00581-52), and has therefore been performed in accordance with the ethical standards established in the 1964 Declaration of Helsinki. After being informed of the various procedures and objectives of the study, all subjects signed a consent form. The subjects were selected according to their absence of excessive diurnal sleepiness measured by the Epworth Sleepiness Scale (score < 10) [29] and their chronotype established on the basis of their answers to the Horne and Ostberg [30] questionnaire. Only the following chronotypes were included in the study: neither morning nor evening (*n* = 11), moderately morning (*n* = 2), and moderately evening (*n* = 6) types. Subjects who were definitively morning or definitively evening types were excluded (*n* = 5) as their circadian rhythms may have been phase-advanced or phase-delayed in comparison to moderate morning or evening types [31]. Therefore, as a result, they may have induced large interindividual variability in the results [32].

### 2.2. Procedure

The subjects took part in two experimental test sessions set up at 6:00 a.m. and 6:00 p.m. in a counterbalanced order. When the subjects were due to take part in the morning test session, they were gathered in the laboratory the night before from 9:30 p.m. to lie down to sleep at 10:30 p.m. The subjects were woken up at 5:00 a.m. in order not only to guarantee a minimum of 6 h in bed, but also to respect a 1 h waking period before the test session [33, 34]. These precautions are generally applied in chronobiological studies in order to avoid the effects of partial sleep deprivation [35] and sleep inertia [36] on measurements carried out in the morning. In addition, no food or drink was consumed before this test session in order to limit interindividual variability observed in cognitive, psychomotor, and physical performances [33, 34].

When the subjects were evaluated at 6:00 p.m., they were instructed to consume the preceding meal at least 3 h before the test session [32] and to come to the laboratory at 3:00 p.m. The consumption of stimulant drinks (coffee, tea, and energy drinks) and participation in physical activities were prohibited in order to avoid their masking effects on diurnal fluctuations in postural control [37]. The mean ambient temperature of the laboratory was 21.9 + 1.8°C during the test sessions.

### 2.3. Measurements

#### 2.3.1. Temperature

Before each test session the subjects were asked to lie down and relax and not to eat or drink anything for 15 min [38]. Then the oral temperature was measured by an experimenter using a digital clinical thermometer (Omron, accuracy: 0.05°C), inserted sublingually for at least 3 min.

#### 2.3.2. Sleepiness/Vigilance

*Sign Cancellation Test.* Vigilance was evaluated by a sign cancellation test [39]. A sheet of paper with 25 lines made up of 20 signs, each distributed at random (i.e., eight different forms for a total of 500 signs), was given to each subject. After sitting at a table in a quiet and neutral room, each subject was instructed to cross out all of the signs that matched three of the eight types present. The three shapes to be crossed out were changed in each test session. Various performance indicators were selected, such as time taken to complete the grid and the total number of errors, which corresponded to both omissions and false alarms (i.e., signs that were incorrectly crossed out). This type of test has been selected because it is particularly sensitive in revealing the effects of time-of-day on vigilance [40].

#### 2.3.3. Posturographic Assessments

The capacity of the subjects to maintain their balance was evaluated using a force platform (PostureWin©, Techno Concept, Céreste, France; 40-Hz frequency, 12-bit A/D conversion) that recorded displacements in the centre of foot pressure (COP) with three strain gauges. The subjects were placed according to precise marks. Their legs were stretched out and their feet formed a 30° angle relative to each other (intermalleolar distance of 5 cm). The test lasted 51.2 s and was first performed with eyes open (EO) and then with eyes closed (EC). In the EO condition, the subjects looked at a fixed level target 90 cm away. In the EC condition, they were asked to keep their gaze straight-ahead without speaking or clenching their teeth.

The COP surface area (90% confidence ellipse) evaluates a subject's postural performance: the smaller the area, the better the performance [41]. The path length (PL), the anteroposterior PL, the mediolateral PL, and the ratio corresponding to the length of the COP displacement according to the surface (LFS: length as a function of surface), index of the energy expenditure [42], and Romberg's index (RI) ((surface EC/surface EO) × 100), which evaluates the contribution of vision to the maintenance of posture [43], were also computed.

Fast Fourier transforms were applied to the COP displacements from 0 to 20 Hz. Hence, the total spectral energy was calculated and distributed between three frequency bands: low frequencies (LF): 0–0.5 Hz; medium frequencies (MF): 0.5–2 Hz; and high frequencies (HF): >2 Hz [44], for the anteroposterior and mediolateral directions [19]. The values of these three frequency bands were expressed as a percentage of the total spectral energy [45]. Low frequencies mostly account for visual and vestibular regulation [1, 46], medium frequencies for cerebellar regulation [45], and high frequencies for the involvement of the reflexive loop [20, 44, 46].

### 2.4. Statistical Analysis

In order to determine possible differences between the data obtained at 6:00 a.m. and at 6:00 p.m., oral temperature and the time to complete the sign cancellation test were analysed using a* t*-test for matched samples.

The total number of errors, the number of errors made by omission, and the number of false alarms that occurred during the sign cancellation test provided quantitative and discontinuous data. A Wilcoxon test for matched samples was applied to the results obtained at 6:00 a.m. and at 6:00 p.m.

Postural sways were analysed by a 2 (time-of-day: 6:00 a.m.* versus* 6:00 p.m.) × 2 (condition of vision: normal* versus* occluded) repeated-measure analysis of variance (ANOVA). Dependent variables were COP surface area, PL, anteroposterior PL, mediolateral PL, and LFS ratio. In addition, the percentage of low-frequency, medium-frequency, and high-frequency bands obtained in the anteroposterior and mediolateral directions during the measurements with eyes open were analysed using a 2 (time-of-day: 6:00 a.m.* versus* 6:00 p.m.) × 3 (frequency band: low, medium, and high) ANOVA. When an interaction effect was observed, a post hoc analysis (least significant difference) was applied. The total spectral energy was measured in the anteroposterior and mediolateral directions separately and Romberg's index was also calculated. The data obtained at 6:00 a.m. were compared to those obtained at 6:00 p.m. using a* t*-test for matched samples.

All differences were regarded as significant at a *P* < 0.05. All statistical analyses were performed using STATISTICA^©^ software (Statsoft, France, version 7.1).

## 3. Results

### 3.1. Oral Temperature

A significant effect of time-of-day was found for oral temperature (*t* = 9.55; *P* < 0.01). The temperature was higher at 6:00 p.m. than at 6:00 a.m. (+0.89°C) (Table 1).

### 3.2. Sleepiness/Vigilance

The subjects were more efficient at the sign cancellation test at 6:00 p.m. than at 6:00 p.m. (*t* = 3.35; *P* < 0.01) while no significant difference was observed concerning the errors made by omission (*z* = 1.44; NS) or false alarms (*z* = 0.59; NS) according to the time-of-day (Table 1).

### 3.3. Posturographic Assessments

A main effect of the condition of vision was observed on all parameters (PL (*F*_(1,18)_ = 133.4; *P* < 0.001), anteroposterior PL (*F*_(1,18)_ = 116.4; *P* < 0.001), mediolateral PL (*F*_(1,18)_ = 30.57; *P* < 0.001), COP surface area (*F*_(1,18)_ = 22.04; *P* < 0.001), and LFS ratio (*F*_(1,18)_ = 6.55; *P* < 0.05)). These parameters were all increased under the EC condition in comparison with the EO condition (Table 1).

The ANOVA indicated a significant effect of time-of-day on COP surface area (*F*_(1,18)_ = 11.84; *P* < 0.01). Regardless of the condition of vision, the COP surface area decreased by 27.5% in the evening compared to the morning values. In addition, time-of-day also had a significant effect on the LFS ratio (*F*_(1,18)_ = 5.02; *P* < 0.05). The average LFS ratio was higher in the evening (+8.4%) compared to the morning values (Table 1).

More importantly, an interaction effect of “time-of-day” × “condition of vision” was observed on PL (*F*_(1,18)_ = 5.90; *P* < 0.05), mediolateral PL (*F*_(1,18)_ = 4.74; *P* < 0.05), and LFS ratio (*F*_(1,18)_ = 5.70; *P* < 0.05). The post hoc analysis indicated that, under the EO condition, PL, mediolateral PL, and the LFS ratio increased from 6:00 a.m. to 6:00 p.m. by 12.8%, 10.9%, and 14.7%, respectively. However, these three parameters were not modified throughout the day under the EC condition. Moreover, it must be noted that Romberg's index did not change throughout the day (*t* = 0.85; NS).

**Figure 1:**
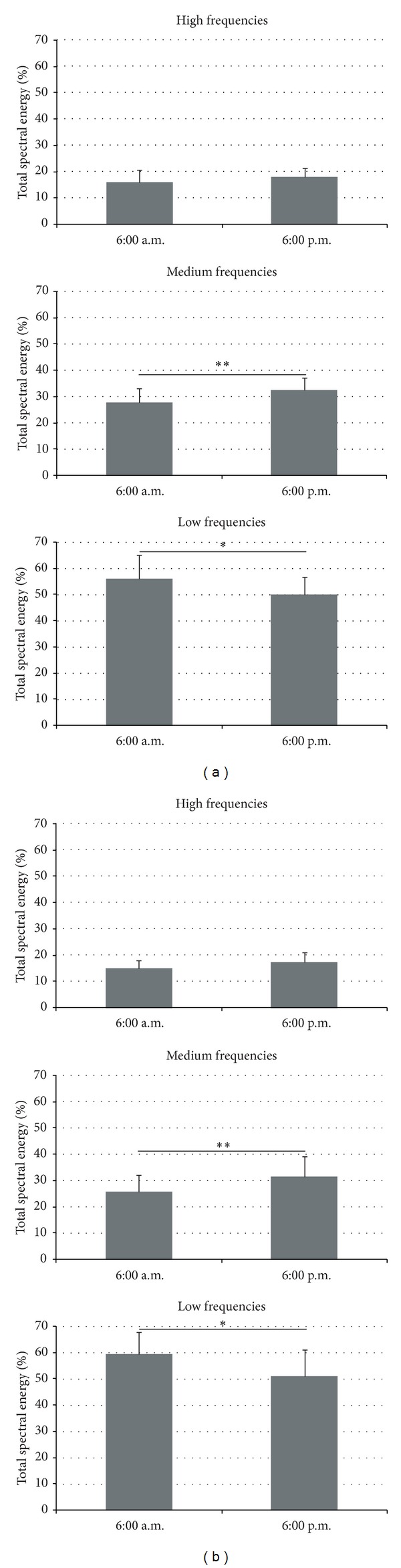
Percentage of total spectral energy in the anteroposterior direction (a) and mediolateral direction (b) measured at 6:00 a.m. and 6:00 p.m. Top: high-frequency band. Middle: medium-frequency band. Bottom: low-frequency band. **indicates a significant difference (*P* < 0.01); *P < 0.05.

The analysis of the total spectral energy in the anteroposterior direction based on the fast Fourier transform showed no significant difference (*t* = −1.66; NS) between 6:00 a.m. (17.20 ± 4.52 mm^2^ *·*Hz^−1^) and 6 : 00 p.m. (18.85 ± 4.47 mm^2^ *·*Hz^−1^). More precisely, the statistical analysis indicated that in the anteroposterior direction the contribution of the low-frequency band (52.9%) was higher than that of the medium-frequency (30.1%) and high-frequency bands (16.9%), regardless of the time-of-day (*F*_(2,36)_ = 128.19; *P* < 0.001). Moreover, an interaction effect of “time-of-day” × “frequency band” was observed (*F*_(2,36)_ = 9.45; *P* < 0.001). The proportion of the low-frequency band decreased (9.61 ± 2.81 mm^2^ *·*Hz^−1^ (i.e., 56.12 ± 8.86%) at 6:00 a.m.* versus *9.36 ± 2.39 mm^2^ *·*Hz^−1^ (i.e., 49.81 ± 6.73%) at 6:00 p.m.), while the medium-frequency band increased (4.79 ± 1.69 mm^2^ *·*Hz^−1^ (i.e., 27.8 ± 5.45%) at 6:00 a.m.* versus *6.13 ± 1.83 mm^2^ *·*Hz^−1^ (i.e., 32.34 ± 4.99%) at 6:00 p.m.). In contrast, the contribution of the high-frequency band (2.80 ± 1.30 mm^2^ *·*Hz^−1^ (i.e., 16.08 ± 4.49%) at 6:00 a.m.* versus *3.40 ± 1.14 mm^2^ *·*Hz^−1^ (i.e., 17.87 ± 3.40%) at 6:00 p.m.) remained stable throughout the day (Figure 1(a)).

In the mediolateral direction, the statistical analysis of the fast Fourier transform revealed no significant difference (*t* = −0.61; NS) in the total spectral energy between 6:00 a.m. (12.09 ± 3.91 mm^2^ *·*Hz^−1^) and 6:00 p.m. (12.61 ± 3.49 mm^2^ *·*Hz^−1^). As in the anteroposterior direction, the contribution of the low-frequency band (55.2%) was higher than that of the medium-frequency (28.6%) and high-frequency bands (16.2%), regardless of the time-of-day (*F*_(2,36)_ = 83.41; *P* < 0.001). More interestingly, the contribution of the various sensory inputs was modified throughout the day (*F*_(2,36)_ = 23.6; *P* < 0.01). The proportion of the low-frequency band, which mostly accounts for visuovestibular regulation, was higher at 6:00 a.m. (7.18 ± 2.58 mm^2^ *·*Hz^−1^ (i.e., 59.35 ± 8.32%)) than at 6:00 p.m. (6.47 ± 2.12 mm^2^ *·*Hz^−1^ (i.e., 51.01 ± 10.12%)). In contrast, the contribution of the medium-frequency band, considered to be an expression of cerebellar regulation, increased from 6:00 a.m. (3.13 ± 1.29 mm^2^ *·*Hz^−1^ (i.e., 25.71 ± 6.56%)) to 6:00 p.m. (3.99 ± 1.51 mm^2^ *·*Hz^−1^ (i.e., 31.55 ± 7.79%)). Finally, the contribution of the high-frequency band, which is sensitive to the involvement of the reflexive loop, remained stable throughout the day (1.79 ± 0.64 mm^2^ *·*Hz^−1^ (i.e., 14.93 ± 3.01%) at 6:00 a.m.* versus *2.20 ± 0.74 mm^2^ *·*Hz^−1^ (i.e., 17.44 ± 3.55%) at 6:00 p.m.) (Figure 1(b)).

## 4. Discussion

In this study, oral temperature, vigilance, and postural sway parameters have been recorded in parallel to specify (i) the link between these parameters and (ii) the role played by vestibular, visual, and somatosensory inputs in the diurnal fluctuations of postural control. The results confirm the presence of diurnal fluctuations in postural control in relation to the increase in body temperature and sleepiness/vigilance levels improvement throughout the day. These diurnal fluctuations in postural control are mainly determined by a decrease in visuovestibular and an increase in cerebellar regulations throughout the day.

The evaluation of postural control during quiet standing indicates a significant effect of time-of-day on COP surface area and LFS ratio. These results are in agreement with those of a preliminary study [7], and they also confirm the idea that postural swaying is greater in the early morning, when the level of sleepiness is higher [6, 22] and body temperature is lower than in the evening [4, 22, 23]. The increase in the LFS ratio in the evening is induced by a significant increase in PL and, in particular, in the mediolateral PL observed under the EO condition. These results were also obtained in previous studies [3, 23], in which lower COP velocities have been reported during morning sessions compared to evening ones after a night of normal sleep. As suggested by Santarcangelo et al. [47], these observations might be due to changes in postural strategies; they argue that a same or even reduced area swept in different conditions; larger LFS values indicate a longer COP trajectory and, thus, a greater number of shorter oscillations. According to a simple model of postural control, increased system stiffness and damping lead to decreases in sway displacement and increases in sway velocity [48]. Changes in postural control strategies during quiet stance involve changes in ankle, hip, or a combination of both [49, 50], which can be observed in postural recordings. An ankle strategy is mainly implicated in regulations in the anteroposterior direction and based on somesthetic input, while a hip strategy contributes to the stabilisation in the mediolateral direction and involves vestibular input [49]. Applying spectral analysis on each direction would bring further information on the sensory inputs underlying these changes in postural strategies.

Considering time-of-day effects on postural sway, the fact that the RI was statistically unchanged in the morning and in the evening confirms that the contribution of visual input is not modified by the hour of the recordings [3, 7]. Therefore, the modifications observed in balance strategies throughout the day are probably due to modifications in the effectiveness of the other sensory systems (vestibular and/or proprioceptive) [51] and/or in the integration of the various sensory inputs via the cerebellum [52]. The decrease during the day in the low-frequency band [53] confirms that the visuovestibular system is highly affected by circadian rhythms and that the sensitivity of visual [54] and especially of vestibular [55] systems may improve throughout the day. As hypothesised by Morad et al. [4], the main submechanisms of postural control may have different thresholds and vulnerabilities to fatigue and sleep deprivation and also to circadian rhythmicity. In fact, our results suggest that the detection thresholds of imbalance situations may be more sensitive in the evening than in the morning. Previous studies have shown strong links between the mechanisms responsible for circadian rhythmicity and the receptors of the vestibular macula [56]. Moreover, the increase in fluid and blood circulation velocity at the cerebral level and, more precisely, in the vestibular system throughout the day [57] acts in parallel with the increase in body temperature [21], which may also contribute to the improvement of sensitivity of the vestibular system. As we observed in this study, this could induce more COP displacements on a reduced surface area (corrective movements). In contrast with the decrease observed in the low-frequency band, the contribution of the medium-frequency band, related to cerebellar regulation [45], was higher in both the anteroposterior and mediolateral directions at 6:00 p.m. than at 6:00 a.m. It seems that cerebellar regulation improves throughout the day in order to regulate postural sway more efficiently, as it requires faster adaptations. Several recent studies have shown that the “hip strategy” is particularly efficient in the maintenance of the centre of body mass (COM) above the support area [58], while minimising muscular and also neural activation [59]. This mechanism may be due to changes in CNS activation [22, 26], as reflected by the results obtained for the sign cancellation test, showing that the subjects were less sleepy in the evening. As for the involvement of the reflexive loop reflected by the high-frequency band [4, 53], our results confirmed those of previous studies reporting diurnal fluctuations in low- and medium-frequency bands with no modifications to the high-frequency band [4, 6]. Since this higher part of the spectrum is the least involved in postural control, it seems that even though nervous conduction velocity [60] and muscular strength [61] are higher around 6:00 p.m. than at 6:00 a.m., the CNS does not ensure compensatory processes by using proprioceptive regulation [3].

**Figure 2:**
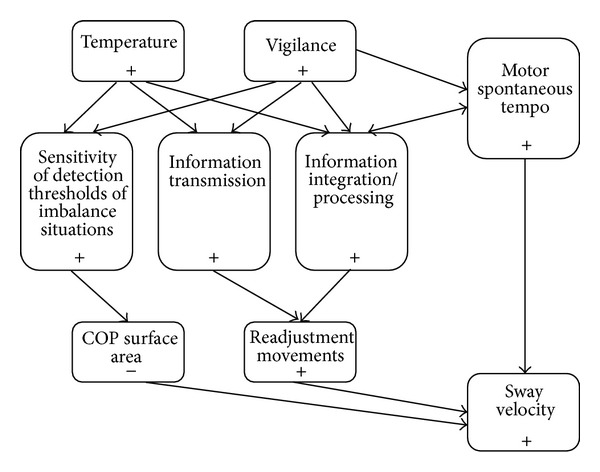
Processes involved in changes in postural strategies between morning and evening measurements. COP surface area: center-of-pressure surface area; +: 6:00 a.m. < 6:00 p.m.; −: 6:00 a.m. > 6:00 p.m.

Given the evolution of the different parameters recorded in this study, various assumptions may be formulated to explain changes in postural strategies between morning and evening measurements (Figure 2).

Firstly, various studies have demonstrated that motor spontaneous tempo increases throughout the day [62], which can also be observed in freely chosen pedal rate [63, 64]. It can be proposed that the cerebellum, considered to be a cerebral structure responsible for movement coordination, may impulse a faster oscillatory frequency throughout the day. Secondly, nervous conduction [60] and the sensitivity of detection thresholds of near-fall situations may be improved in the evening, which may explain the decrease in surface area when temperature and vigilance levels are higher. Thirdly, as the reflexive loop is the least involved in postural regulation and muscular strength is not required to ensure balance, the CNS would not focus on this path to improve stability. All these observations coincide with the distribution of postural sway frequencies, indicating an increase in cerebellum regulation, probably due to an improvement of the sensitivity of detection thresholds of near-fall situations. This increase is in contrast with a disengagement of the visuovestibular contribution.

Although our work addresses an interesting question by examining which postural control mechanisms underlie the observed diurnal fluctuations, several issues should be considered before firm conclusions can be drawn. Firstly, only two time schedules have been selected to observe the diurnal fluctuation of postural control. However, even if more time points would have given precise information on the relationship between postural control and temperature and/or vigilance, the times of testing (6:00 a.m. and 6:00 p.m.) were chosen close to the expected bathyphase and acrophase and to allow the observation of maximal diurnal fluctuations [7]. In addition, various precautions were respected to limit potential confounding variables when studying time-of-day effects on postural control [5]. Secondly, the subjects spent 6 hours in bed, which could be considered as not sufficient to recover completely. Depending on the study under consideration, 6 to 9 hours are recommended [65]. Moreover, this experimental methodology is extensively used in chronobiological studies [32], and it has been confirmed that only one night with an early awakening does not influence the observation of diurnal fluctuations [66]. Thirdly, the subjects were awoken one hour before testing, which allowed sleep inertia effects to dissipate [36]. It has also been shown that many cognitive, psychomotor, and physical performances do not differ significantly between measurements carried out at 6:00 a.m. after being awakened either at 4:00 a.m. or at 5:00 a.m., that is, 1 or 2 hours before the test session [33, 34].

To conclude, the results of this study confirm that postural control is more effective in the evening, during the acrophase of the body temperature rhythm, and also when subjects feel less sleepy and more vigilant than they do in the early morning. Different strategies of postural control occur depending on time-of-day. It seems that in the morning there are fewer but extended COP displacements, whereas in the evening there are more short-length COP displacements in a reduced surface. Analysis of spectral content of postural sway bidirectionally indicates that these adaptations are probably induced by a decrease in visuovestibular regulation, in contrast with an improvement of cerebellar regulation, which are dependent on both the increase in body temperature and CNS activation. These results may also have direct application in terms of rehabilitation. Practitioners should focus on imbalanced situations with a higher frequency of readjustments in the evening to induce significant progresses.

## Figures and Tables

**Table 1:** Oral temperature, sign cancellation test, and posturography parameters in subjects under eyes open (EO) and eyes closed (EC) conditions at 6:00 a.m. and 6:00 p.m. (mean ± SD; *n* = 19).

		6:00 a.m.	6:00 p.m.
Oral temperature (°C)		35.99 ± 0.32	36.77 ± 0.22*

Sign cancellation test (s.)		340.36 ± 62.46	287.01 ± 53.41*

COP surface area (mm^2^)	EO	120.73 ± 62.89	89.03 ± 40.21*
	EC	179.76 ± 86.52	128.89 ± 60.43*

PL (mm)	EO	375.28 ± 117.34	423.49 ± 107.28*
	EC	539.86 ± 143.57	539.28 ± 149.04

Mediolateral PL (mm)	EO	188.23 ± 63.09	208.91 ± 54.59*
	EC	251.64 ± 78.33	247.95 ± 68.87

Anteroposterior PL (mm)	EO	284.25 ± 90.96	316.07 ± 88.32
	EC	424.58 ± 114.18	420.51 ± 130.14

LFS ratio	EO	0.86 ± 0.28	0.98 ± 0.22*
	EC	0.96 ± 0.26	0.99 ± 0.24

Romberg's index		168.54 ± 84.14	150.67 ± 50.46

COP surface area: centre-of-pressure surface area; PL: path length; anteroposterior PL: anteroposterior path length; lateral PL: lateral path length; LFS ratio: path length as a function of surface; Romberg's index: ratio between the centre-of-pressure surface areas measured under the eyes open (EO) and eyes closed (EC) conditions. ∗Significant difference between 6:00 a.m. and 6:00 p.m.
